# The effects of ectomycorrhizal and saprotropic fungi on soil nitrogen mineralization differ from those of arbuscular and ericoid mycorrhizal fungi on the eastern Qinghai-Tibetan Plateau

**DOI:** 10.3389/fpls.2022.1069730

**Published:** 2023-01-04

**Authors:** Miaomiao Zhang, Shun Liu, Xiangwen Cao, Miao Chen, Jian Chen, Gexi Xu, Zuomin Shi

**Affiliations:** ^1^ Key Laboratory of Forest Ecology and Environment of National Forestry and Grassland Administration, Ecology and Nature Conservation Institute, Chinese Academy of Forestry, Beijing, China; ^2^ Miyaluo Research Station of Alpine Forest Ecosystem, Lixian County, Sichuan, China; ^3^ Co-Innovation Center for Sustainable Forestry in Southern China, Nanjing Forestry University, Nanjing, China; ^4^ Institute for Sustainable Plant Protection, National Research Council of Italy, Torino, Italy

**Keywords:** net N mineralization, mycorrhizal associations, soil fungi, soil enzymes, microclimate

## Abstract

Interactions between soil fungi and soil environmental factors regulate soil nitrogen (N) mineralization rates on the eastern Qinghai-Tibetan Plateau. Some studies have also illuminated differences in soil N mineralization rate based on different mycorrhizal forests, but the associated effect of soil fungal functional guilds and soil environmental factors underlying this process are not well-understood. Three primary forests respectively dominated by *Abies fargesii* var. *faxoniana* (ectomycorrhizal, EcM), *Cupressus chengiana* (arbuscular mycorrhizal, AM) and *Rhododendron phaeochrysum* (ericoid mycorrhizal, ErM) trees were selected in this area. Meanwhile, soil net N mineralization rate, soil fungal composition and soil enzyme activity among these three mycorrhizal forests were studied. Our results showed that there were significant differences in the seasonal variation of soil net N mineralization rates among three mycorrhizal forests. Soil net N mineralization rate in the AM forest was faster. EcM fungi and saprotroph are the main functional guilds in these three mycorrhizal forests. Meanwhile, the relative abundances of soil fungal functional guilds, soil temperature and soil peroxidase activity could explain 85.0% in the difference of soil net ammonification rate among three mycorrhizal forests. In addition, soil temperature, soil water-filled pore space and soil ammonium content play a central role in controlling the differing soil net nitrification rate among three mycorrhizal forests. Our results suggest differences in soil net mineralization among different mycorrhizal forest types are driven mainly by soil net ammonification. Soil fungal functional guilds and temperature regulate the rate of soil net ammonification by modulating soil peroxidase activity.

## Introduction

Many ecological properties of forests are affected by soil nitrogen (N) uptake and utilization, including plant growth, interspecific competition and soil carbon (C) sequestration (Cole et al., 2008; Levy-Booth et al., 2014). Forest soil N is present in two organic forms: minerals and organic compounds (Adamczyk et al., 2016). However, plants absorb mainly inorganic N (e.g., ammonium $\left(NH_{4}^{+}-N\right)$ , nitrate $\left(NO_{3}^{-}-N\right)$ ) (Zhang et al., 2018), with a minor amount of low molecular weight organic N being absorbed under special environmental conditions (Li et al., 2019). Soil organic N is only absorbed easily by plants after its transformation into inorganic N by soil microbial mineralization (Liu et al., 2017). Therefore, soil N mineralization is a critical process which determines soil N availability and ecosystem primary productivity (Li et al., 2019).

Soil fungi are the primary decomposers of organic matter and driver of nutrient cycling in forest ecosystem (Zeilinger et al., 2016). Soil fungi in forest are broadly classified into two functional groups: free-living saprotrophs and plant root symbiotic fungi (Bödeker et al., 2016). Fungi that live in symbiotic relationships with plant roots may do so through ectomycorrhizal (EcM) or other types of symbiosis, including arbuscular mycorrhizal (AM) and ericoid mycorrhizal (ErM) (Hobbie and Högberg, 2012; Tedersoo et al., 2020; Wang et al., 2022). The differences among these three groups of mycorrhizal fungi are important because of their biogeochemical significance. Specific ecological functions of different mycorrhizal fungi as well as complex interactions between mycorrhizal fungi and other soil microbes might influence the activity and abundance of N cycling functional guilds (Tedersoo et al., 2020; Saifuddin et al., 2021), resulting in changes to N cycling among different mycorrhizal forests (Bahram et al., 2020). Compared to EcM forest or ErM forest, soil N cycling in AM forest is often more ‘rapid’ and ‘open’ which is dominated by inorganic N cycling patterns (Phillips et al., 2013; Tedersoo and Bahram, 2019). Both EcM and ErM fungi could produce a wide range of enzymes (such as oxidases and peroxidases) that enable them to release N from soil organic matter (Orwin et al., 2011; Ward et al., 2021). By contrast, AM fungi are much less capable of producing these enzymes, typically lacking the complement of enzymes that decompose organic matter (Saifuddin et al., 2021). Accordingly, AM plants are primarily responsible for absorbing inorganic N in exchange for carbon derived from plant photosynthesis (Johnson, 2010; Van der Heijden et al., 2015; Han et al., 2020; Babalola et al., 2022), whereas EcM and ErM plants typically obtain more organic N from soil (Wurzburger and Hendrick, 2009). Given that EcM and ErM fungi have broader enzymatic capabilities, they could compete directly with saprotrophs for organic substrates (Adamczyk et al., 2016). This interspecific competition could decrease rates of soil N mineralization in forests (Argiroff et al., 2022). Yet the no-inhibited saprotrophs in AM forest soil could enhance litter decomposition to accelerate soil N mineralization (Midgley and Phillips, 2016; Saifuddin et al., 2021). Previous studies also had shown that eliminating AM fungi from soil could slow soil N mineralization by reducing the substrate supply of saprotrophs (Cheng et al., 2012; Averill et al., 2014). Similarly, Bahram et al. (2020) indicated that the relative abundances of AM fungi, saprotrophs and pathogens were all higher in AM forest than EcM forest. These alterations in microbial composition reflect the rapid nutrient cycling of AM forest soil.

In addition, the soil N mineralization rate features high spatial and temporal heterogeneity among different mycorrhizal forests, it being also affected by soil temperature, soil moisture and soil physicochemical properties (Liu et al., 2017). Recent research has revealed that whereas soil moisture and temperature were the main factors impacting the relative abundance of EcM fungi, soil physicochemical properties are the main factors controlling the relative abundance of saprotrophs (Wang et al., 2019). Soil fungal associations that catalyzed organic matter mineralization responded differently to various soil environmental factors and this might lead to uncertainty in soil N mineralization (Mushinski et al., 2020). Although soil fungi and environmental factors among different mycorrhizal forests arguably have certain effects on soil N mineralization, our knowledge of direct and indirect effects of soil fungi and environmental factors on that process is still quite limited.

The eastern Qinghai-Tibetan Plateau presents a unique natural environment, one that contributes critically to soil and water conservation, climate regulation, and biodiversity protection (Wang et al., 2007). It is an important ecological barrier in the middle and upper reaches of the Yangtze River (Chen, 2019). In addition, this area is abundant in natural resources, among which *Abies fargesii* var. *faxoniana*, *Cupressus chengiana* and *Rhododendron phaeochrysum* are important forest trees (Feng et al., 2017; Du et al., 2021), which respectively are the typical EcM, AM and ErM trees (Soudzilovskaia et al., 2020). Previous studies in this area have partially explored the changes of soil fungal community structure and soil N mineralization among different forest types (Liu et al., 2021; Chen et al., 2022). Yet the potential mechanism of the difference of soil N mineralization among different mycorrhizal forests remains to be further studied. In order to elucidate the dominant factors responsible for differential soil N mineralization among three mycorrhizal forests. Seasonal variation of soil net N mineralization rate, soil fungal functional guilds composition and soil enzyme activity among these three mycorrhizal forests (EcM, AM and ErM forests) were measured. We hypothesized that (1) the soil net N mineralization rate was the highest in AM forest, (2) the relative abundance of EcM fungi and AM fungi was relatively higher in EcM and AM forests, respectively, (3) synergy between soil fungal functional guilds and soil environmental factors could drive the activity of soil oxidases and peroxidases, and (4) soil oxidases and peroxidases activity have a greater effect on soil net ammonification than soil net nitrification.

## Materials and methods

### Site description and design

This study was conducted in in the upper reaches of the Minjiang River, western Sichuan Province (31°35′~31°53′ N, 102°2′~102°48′ E), which is located in the outermost part of the fold belt on the eastern Qinghai-Tibetan Plateau (Xu et al., 2021). Its altitude ranges from 2,200 to 5,500 m. The climate with an average annual temperature of 2~4°C, the highest temperature is 23.7°C in summer, and the lowest temperature is -18.1°C in winter. Annual precipitation is 700~1000 mm and concentrated mainly in the growing season (Chen, 2019). The soil in this area is defined as mountain brown soil, mountain brown cinnamon soil and subalpine meadow soil according to the Chinese soil taxonomic classification (Liu, 2010; Feng et al., 2017; Chen, 2019).

Three different primary forests with different mycorrhizal types were selected, including *Abies fargesii* var. *faxoniana* primary forest (EcM forest), *Cupressus chengiana* primary forest (AM forest) and *Rhododendron phaeochrysum* primary forest (ErM forest) under similar soil and climate conditions. Eight 15 m ×15 m sample plots (≥ 90% the dominant species by basal area in each sample plot) for each forest type were randomly set. In each forest type, the distance between any two sample plots was more than 50 m.

### Soil sampling and analysis

The mineral soil (0-10 cm) samples were collected from the four corners and center of each plot with soil drill from May to November 2019. Five mineral soil samples were mixed into a zipper storage bag and transported to laboratory in an icebox within 3 h. Meanwhile, five polyvinyl chloride collar cores (PVC cores, 15 cm in height and 5 cm in diameter) were buried into depth of 10 cm in the vicinity of each soil sampling location (Idol et al., 2003). In order to separate water and allow gas movement, the top of the PVC core was covered with a permeable plastic film and its bottom covered with gauze (Liu et al., 2021). The difference between soil $NH_{4}^{+}-N$ and $NO_{3^{-}}-N$ contents each month was used to quantify the rates of soil net ammonification, net nitrification and net N mineralization (Wang et al., 2017), which was the ideal time to estimate changes in soil N (Becker et al., 2015). For the measurement of soil water-filled pore space (WFPS), we followed the methodology of Wang et al. (2010). The temperature of mineral soil was measured with a soil temperature detector in each plot (Liu et al., 2021). Furthermore, the mixed mineral soil was sifted through a 2-mm sieve and gravel and fine roots were removed (Xu et al., 2021). The fresh mineral soil was separated into two parts: one was dried naturally to measure soil pH, soil organic C (SOC) and soil total N (TN); the other was stored at -20°C to analysis enzyme activity and microbial community structure.

Soil $NH_{4}^{+}-N$ and $NO_{3^{\;}-}-N$ contents were quantified using an automatic flow injection analyzer (FIAstar 5000 Analyzer, Sweden). Soil pH was determined using the glass electrode meter method, by setting the 1:2.5 (w/v) ratio of soil material to deionized water. The SOC content was measured by applying the wet oxidation method with K_2_Cr_2_O_7_ and H_2_SO_4_, and FeSO_4_ titration. Soil TN content was determined by the Kjeldahl method (Liu et al., 2020). The soil C: N ratio was calculated as the ratio of SOC to soil TN. Finally, the potential activity of peroxidase (PER) and phenol oxidase (POX) which could drive N from soil organic matter (Jian et al., 2016), were determined *via* microplate fluorescence and photometry (Zheng et al., 2019).

### Molecular and bioinformatics analysis

Samples from May, July and November 2019 were selected for soil fungal functional guilds composition analysis. DNA was extracted from 0.25 g of each soil sample by using the PowerSoil^®^ Kit(100) and the concentration of extracted DNA was then determined by a NanoDrop 1000 spectrophotometer. Soil fungi were amplified fungal ITS2region and selected primers pairs fITS7 (5’- GTGARTCATCGAATCTTTG-3’) and ITS4 (5’-AGCCTCCGCTTATTGATATGCTTAART-3’) (Xiong et al., 2021). The 25 μL PCR reaction mixture contained 8.5 μL of sterile deionized water, 0.75 μL of each primer, 12.5 μL of KAPA Polymerase and 2.5 μL of diluted template DNA. The PCR amplification as followed: begin with 3 min of incubation at 95 °C, then 35 cycles of 98 °C for 30 s, 56 °C for 30 s, 72 °C for 30 s, with a final extension at 72 °C for 10 min. Sterile deionized water was used as a template (negative controls) to determine whether there was contamination in our experiment (Yao et al., 2019). Three PCR reactions were pooled for each sample to reduce random of the PCR reaction (Bödeker et al., 2016). In addition, the PCR products from each soil sample were purified by the Product Gel Purification Kit. An equal quality of purified PCR product from each sample was pooled in and adjusted to 10 ng μL^-1^ (Dong et al., 2021). Sequencing was carried out on an Illumina MiSeq PE platform at the Chengdu Institute of Biology, Chinese Academy of Sciences, China.

The chimeras present in the origin sequencing data were removed using the UNITE database, after which the non-chimeric sequence was quality filtered using Usearch. Remaining sequences were clustered into operational taxonomic units (OTUs) at a 97% sequence similarity. Representative fungal sequences were classified using sintax in the UNITE database with a 0.65 confidence threshold (Edgar, 2016). Finally, the number of sequences per sample was normalized to the smallest sample size using the sub.sample function in MOTHUR (Yao et al., 2019) and then we got 10,952 high-quality reads from 72 soil samples. Soil fungal rarefaction curves (**Figures S1**) were analyzed by R 4.1 software (using the ‘vegan’ package). Soil fungal functional guilds were assigned using the FUNGuild command in Python 3.7 software (Tedersoo et al., 2014; Nguyen et al., 2016). We excluded OTUs that did not belong to a confidence ranking with “probable” or “highly probable” and that were above the genus level (Pfennigwerth et al., 2018).

### Statistical analysis

A two-way ANOVA were used to analyze the effects of forest type, month and their interaction on the changes in soil temperature and soil WFPS. Likewise, we also used this method to analyze differences in the rates of soil net ammonization, soil net nitrification and soil net N mineralization (Liu et al., 2021). For multiple comparisons, Tukey’s HSD method was used. For a given month, differences in soil properties, soil net ammonization rate, soil net nitrification rate, and soil net N mineralization rate and soil enzyme activities among three mycorrhizal forests were performed by a one-way ANOVA.

Pearson correlations were used to analyze the effects of soil enzyme activity, edaphic variables and soil fungal functional guilds classified by FUNGuilds on soil net ammonization rate and soil net nitrification rate among three mycorrhizal forests. Further, the significant influencing factors (*P <* 0.05) after the correlation analysis, soil net nitrification rate and soil net ammonification rate were selected for a structural equation (SEM) analysis. Model estimation was achieved based on the maximum likelihood method. The adequacy of model fit were determined by non-significant χ2 tests (*P* > 0.05), comparative fit index (CFI) (values ≥ 0.9) and standardized root mean square residual (SRMR) (values < 0.08) (Ni et al., 2022).

The SEM analysis only selected the data from May, July and November 2019. The ANOVAs and Pearson correlations were carried out in SPSS 26 software. The SEM analysis was performed using R 4.1 software (using the ‘lavaan’ package). Figures were created by Origin 8.0 software.

## Results

### Edaphic variables

**Figure 1:**
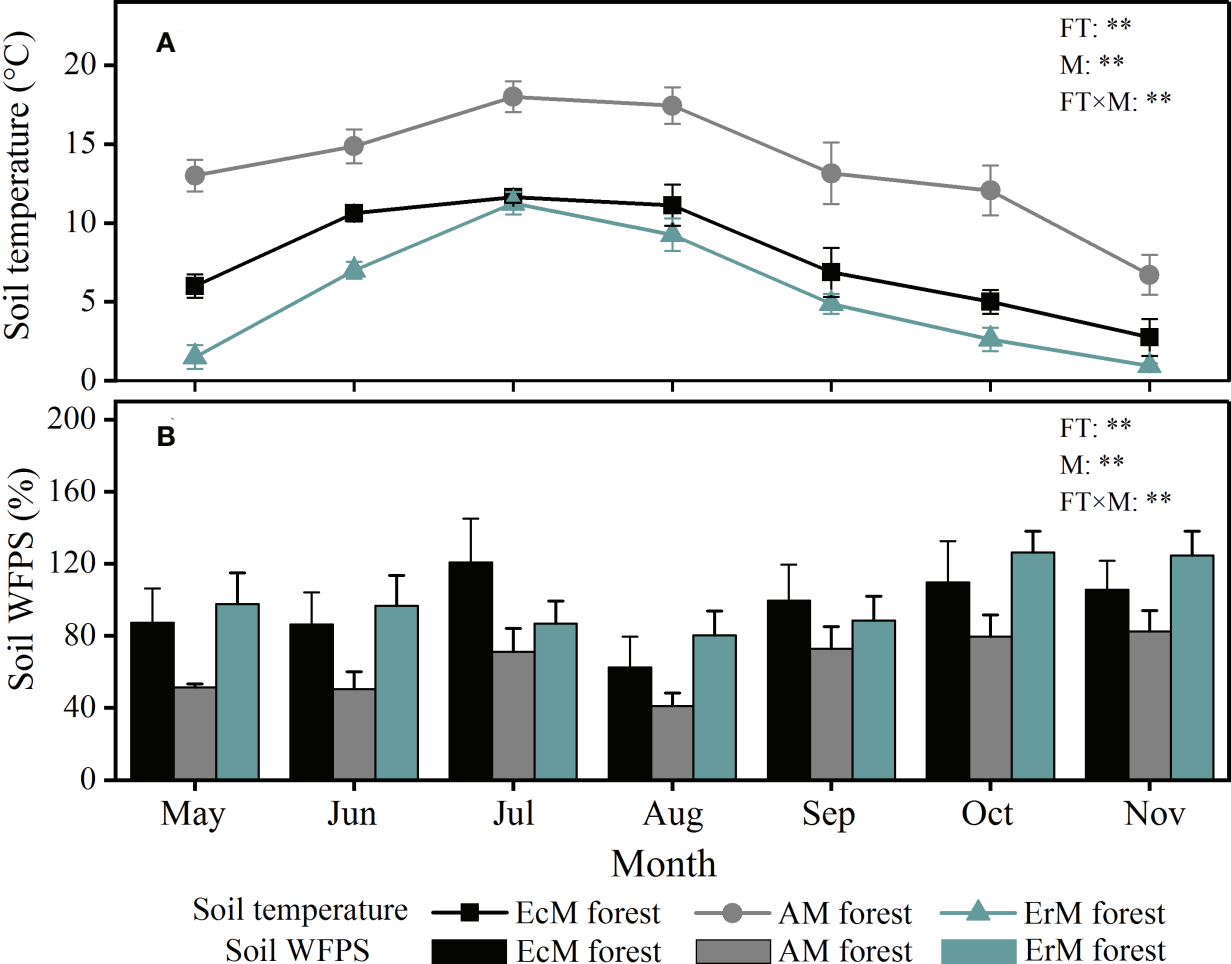
Monthly variations in soil temperature **(A)** and soil WFPS **(B)** among three mycorrhizal forests. The vertical bars are the standard error, n=8. EcM forest, *Abies fargesii* var. *faxoniana* primary forest; AM forest, *Cupressus chengiana* primary forest; ErM forest, *Rhododendron phaeochrysum* primary forest. WFPS, water-filled pore space; FT, forest type; M, month; FT×M, the interaction of forest type and month, **, *P* < 0.01.

Seasonal variation of soil temperature and soil WFPS among three mycorrhizal forests showed significant differences during the study period (*P <* 0.01) (**Figure 1**). Soil temperature of each mycorrhizal forest followed a single peak curve, being highest in July and lowest in November. Soil temperature was the highest in AM forest (6.7-18.0 °C), followed by EcM forest (2.8-11.6 °C) and ErM forest (1.0-11.3 °C) (**Figure 1A**). Contrary to soil temperature, soil WFPS in AM forest (50.46%-82.56%) was significantly lower than that in EcM forest (62.41%-120.68%) and ErM forest (80.23%-126.30%) (**Figure 1B**).

As shown in **Table 1**, there were significant differences in soil pH and soil TN content, both being highest in AM forest (*P <* 0.05). SOC content and soil $NH_{4}^{+}-N$ content were significantly lower in EcM forest than those in AM forest and ErM forest (*P *<0.05). Meanwhile, AM forest had the highest soil $NO_{3^{-}}-N$ content but there was no significant difference between EcM forest and ErM forest. However, soil C: N was significantly lower in AM forest than the other two forests (*P <* 0.05).

**Table 1:** Soil properties among three mycorrhizal forests (values are the means ± 1SE, n = 56).

	EcM forest	AM forest	ErM forest
pH (H_2_O)	5.33 ± 0.48b	7.01 ± 0.56c	5.01 ± 0.28a
SOC (g kg^-1^)	53.32 ± 19.11a	73.23 ± 19.71b	65.31 ± 15.65b
TN (g kg^-1^)	3.57 ± 1.02a	6.14 ± 1.53c	4.81 ± 0.37b
C: N	14.94 ± 1.90b	11.93 ± 2.30a	13.59 ± 1.90b
$NH_{4}^{+}-N$ (mg kg^-1^)	4.70 ± 1.78a	10.66 ± 4.84b	9.25 ± 2.24b
$NO_{\;}3^{-}-N$ (mg kg^-1^)	0.64 ± 0.47a	4.41 ± 2.45b	0.61 ± 0.42a

EcM forest, *Abies fargesii *var*. faxoniana *primary forest*; *AM forest, *Cupressus chengiana *primary forest; ErM forest*, Rhododendron phaeochrysum* primary forest. Lowercase letters (a, b and c) indicate significant differences among three mycorrhizal forests (*P* < 0.05).

### Soil net ammonification, net nitrification and net N mineralization

**Figure 2:**
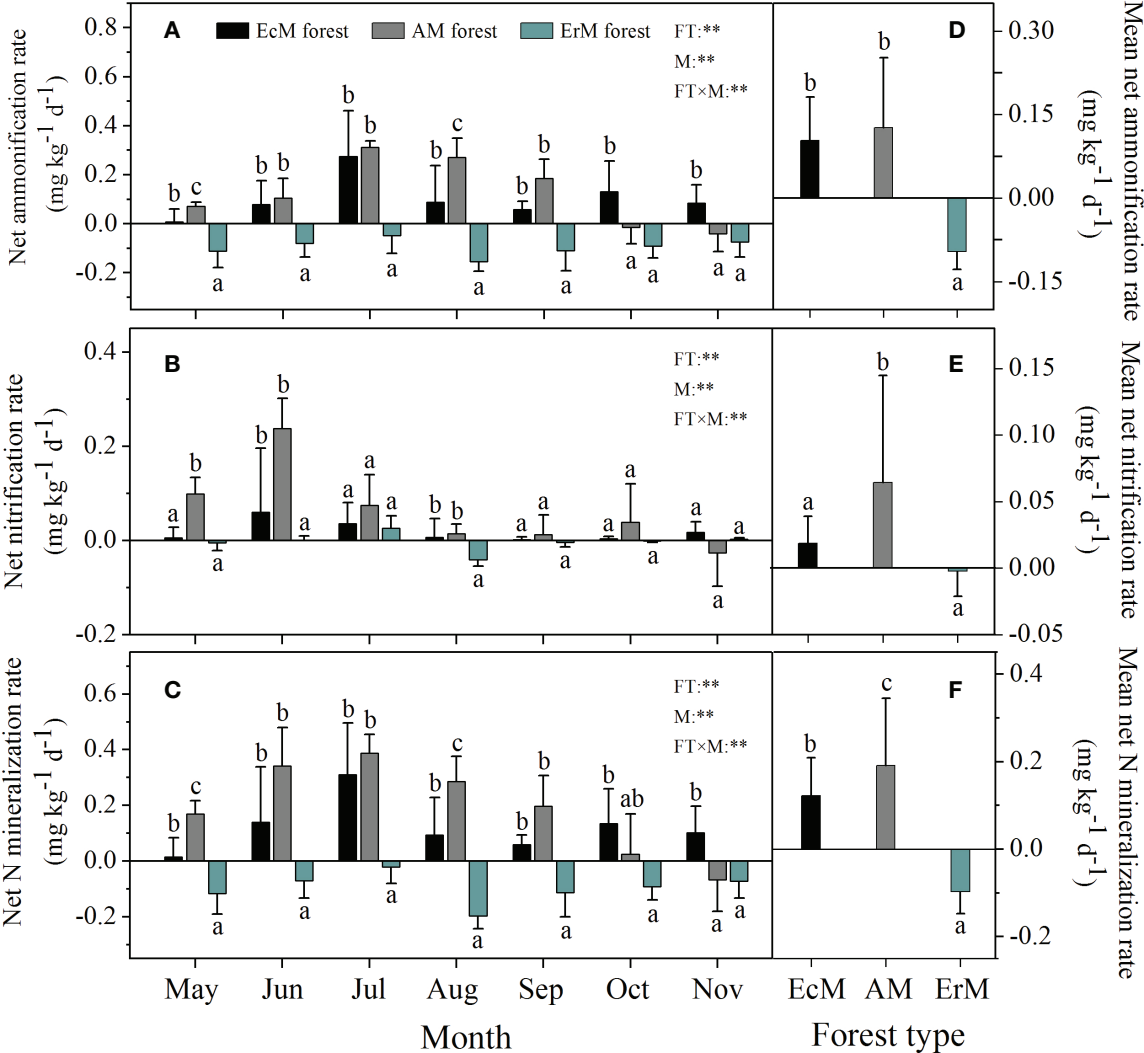
Soil net ammonification rate **(A)**, soil net nitrification rate **(B)**, soil net N mineralization rate **(C)** varied monthly and their mean rates **(D–F)** among three mycorrhizal forests. Data and error bars are the means and standard errors respectively, n=8. EcM forest, *Abies fargesii* var. *faxoniana* primary forest; AM forest, *Cupressus chengiana* primary forest; ErM forest, *Rhododendron phaeochrysum* primary forest. FT, forest type; M, month; FT×M, the interaction of forest type and month. **, *P* < 0.01. Lowercase letters (a, b and c) indicate significant differences among three mycorrhizal forests (*P* < 0.05).

Forest type, sampling month and their interaction had significant effects on soil net ammonization rate (net R_a_), soil net nitrification rate (net R_n_) and soil net N mineralization rate (net N_min_) (*P <* 0.01) (**Figure 2**). Soil net R_a_ ranged from -0.16 to 0.31 mg kg^-1^ d^-1^ among three mycorrhizal forests, with the highest rates occurring in July (**Figure 2A**). Soil mean net R_a_ in ErM forest was negative (-0.09 mg kg^-1^ d^-1^) being also significantly lower than that in EcM forest (0.10 mg kg^-1^ d^-1^) and AM forest (0.13 mg kg^-1^ d^-1^) (*P <* 0.05) (**Figure 2D**).

The significant effect of mycorrhizal forest types on soil net R_n_ was detected in May, June and August. Soil net R_n_ in EcM forest (0.06 mg kg^-1^ d^-1^) and AM forest (0.24 mg kg^-1^ d^-1^) peaked in June while that in ErM forest (0.03 mg kg^-1^ d^-1^) peaked in July (**Figure 2B**). Overall, AM forest had the highest soil mean net R_n_ (0.06 mg kg^-1^ d^-1^) whereas it did not differ significantly between EcM and ErM forests (**Figure 2E**).

The trends in monthly variation of soil net N_min_ were similar to those of soil net R_a_ among three mycorrhizal forests (**Figure 2C**). There were significant differences in soil mean net N_min_ among AM forest (0.19 mg kg^-1^ d^-1^), EcM forest (0.12 mg kg^-1^ d^-1^) and ErM forest (-0.10 mg kg^-1^ d^-1^) (*P <* 0.05) (**Figure 2F**).

### Soil fungal composition and enzyme activity

**Figure 3:**
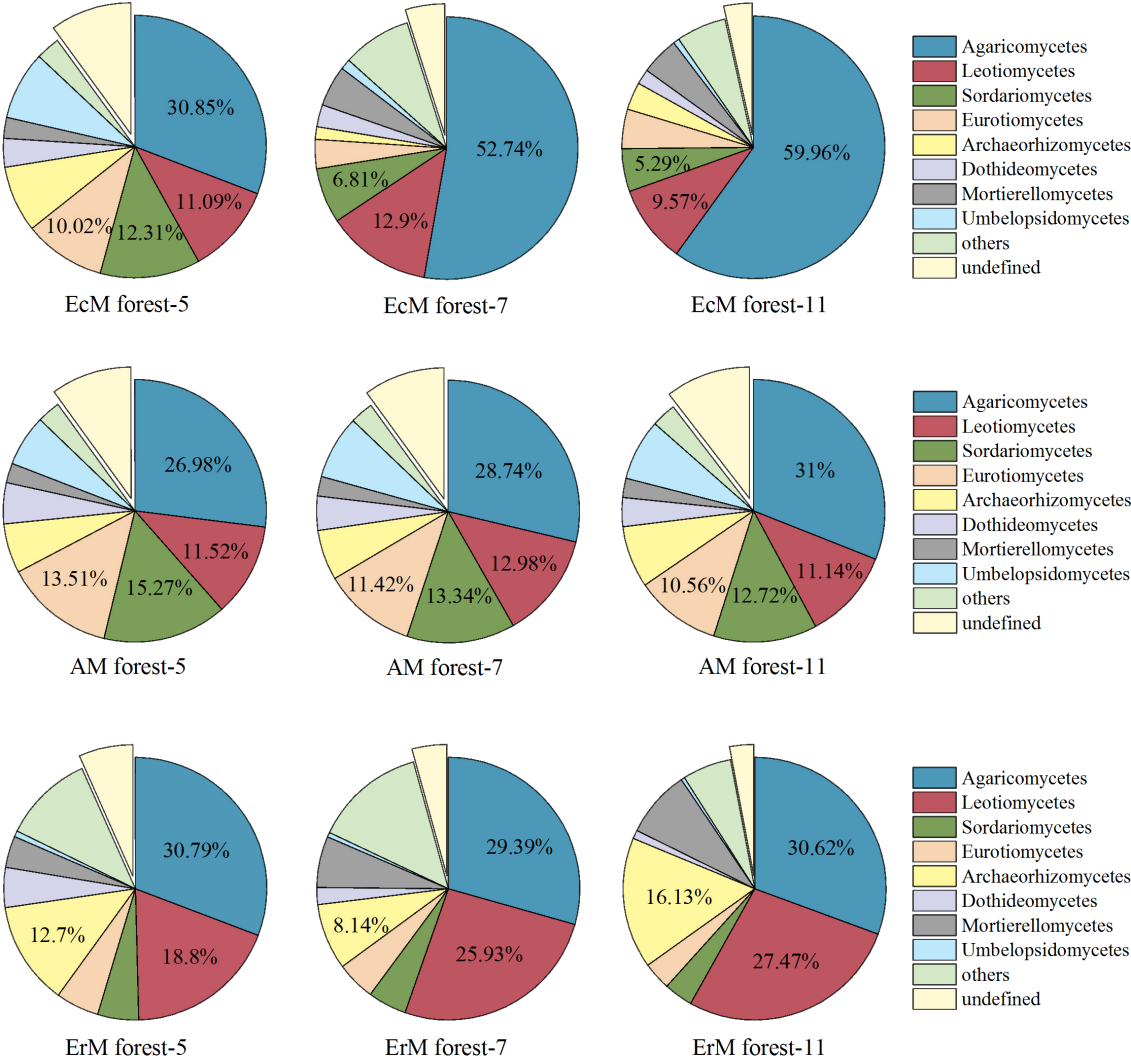
Relative abundances of soil fungal at the class level among three mycorrhizal forests in each different sampling month. ‘Others’ represent the sum of all classes with the relative abundance less than 1%. EcM forest, *Abies fargesii* var. *faxoniana* primary forest; AM forest, *Cupressuschengiana* primary forest; ErM forest, *Rhododendron phaeochrysum* primary forest; -5, -7 and -11 represented May, July and November respectively.

In general, 53 fungal classes were identified by excluding the unidentified fungal groups from the 72 soil samples. The top five dominant classes of soil fungi were Agaricomycetes (26.98%-59.96%), Leotiomycetes (9.57%-27.47%), Sordariomycetes (3.55%-15.27%), Eurotiomycetes (3.46%-13.51%) and Archaeorhizomycetes (1.53%-16.13%). By contrast, Dothideomycetes (1.08%-5.06%), Mortierellomycetes (2.36%-8.25%) and Umbelopsidomycetes (0.47%-8.44%) presented low relative abundances in the three forests (**Figure 3**).

**Figure 4:**
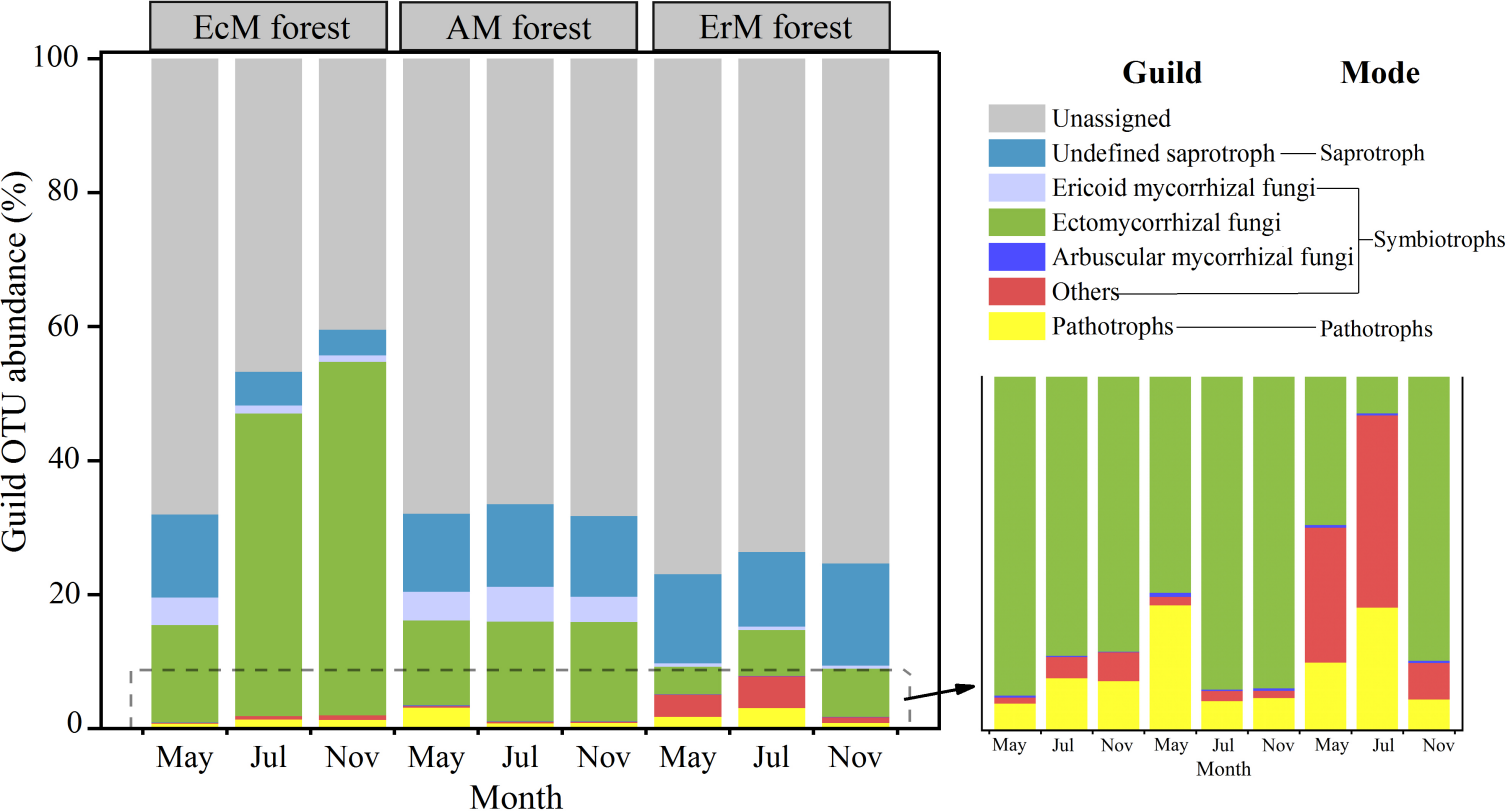
Composition of soil fungal functional guilds among three mycorrhizal forests in each different sampling month. EcM forest, *Abies fargesii* var. *faxoniana* primary forest; AM forest, *Cupressus chengiana* primary forest; ErM forest, *Rhododendron phaeochrysum* primary forest. ‘Others’ include endophyte and lichenized. Pathotrophs include animal pathogen, plant pathogen and mycoparasites. The lower right corner is a partial enlargement.

We also obtained three main trophic modes (saprotroph, symbiotroph and pathotroph) and 10 soil fungal functional guilds from the 10952 OTUs. The relative abundance of the unassigned group (40.49%-77.01%) was dominant in our study. Among the assigned OTUs, the relative abundance of EcM fungi (4.14%-52.80%) was the highest, followed by the undefined saprotroph (SAP) (3.80%-15.21%) and ErM fungi (0.48%-5.18%). The relative abundances of the remaining guilds were lower (**Figure 4**).

**Figure 5:**
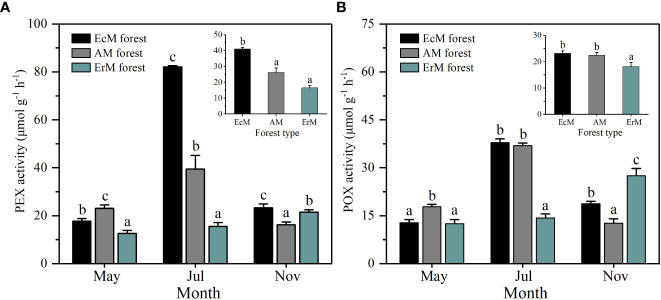
Soil PEX activity **(A)** and soil POX activity **(B)** among three mycorrhizal forests in each different sampling month (lower panel) and means (upper panel). Data and error bars are the means and standard errors respectively, n=8. EcM forest, *Abies fargesii* var. *faxoniana* primary forest; AM forest, *Cupressus chengiana* primary forest; ErM forest, *Rhododendron phaeochrysum* primary forest. PEX, peroxidase; POX, phenol oxidase. Lowercase letters (a, b and c) indicate significant differences among three mycorrhizal forests (*P* < 0.05).

Soil PEX and POX activity each differed significantly among three mycorrhizal forests in each month (*P *< 0.05). Their activity in AM forest and EcM forest peaked in July, while those in ErM forest peaked in November (**Figure 5**). In general, soil mean PEX activity of EcM forest (41.04 μmol g^-1^ h^-1^) was significantly higher than the other two forests (*P *< 0.05) (**Figure 5A**). Soil mean POX activity in ErM forest (18.10 μmol g^-1^ h^-1^) was significantly lower than in EcM forest (23.13 μmol g^-1^ h^-1^) and AM forest (22.46 μmolg^-1^ h^-1^) (*P *< 0.05) (**Figure 5B**).

### Main factors affecting on soil net N mineralization

The results showed that soil net N_min_ of the three mycorrhizal forests were affected differently by soil temperature and soil WFPS. Soil temperature and soil WFPS had no significant differences on soil net R_n_ in EcM forest, but soil net R_a_ in EcM forest was positively correlated with soil WFPS. Both soil net R_a_ and net R_n_ in AM forest were positively correlated with soil temperature but negatively correlated with soil WFPS. In contrast, soil net R_a_ and soil net R_n_ in ErM forest were positively correlated with soil WFPS (**Table 2**).

**Table 2:** Correlations between soil net ammonification rate (net R_a_), soil net nitrification rate (net R_n_) and microclimate: soil temperature (T) and soil WFPS (water-filled pore space).

		EcM forest		AM forest		ErM forest	
Independent variable	T	R	P	R	P	R	P
variable	Net R_a_	0.25	ns	0.77	**	-0.21	ns
	Net R_n_	0.23	ns	0.36	**	-0.04	ns
Independent variable	WFPS	PCCs	P	PCCs	P	PCCs	P
variable	Net R_a_	0.41	**	-0.28	*	0.37	*
	Net R_n_	0.19	ns	-0.43	**	0.34	*

R, Pearson correlation coefficients; P, significance. EcM forest, *Abies fargesii* var. *faxoniana* primary forest; AM forest, *Cupressus chengiana* primary forest; ErM forest, *Rhododendron phaeochrysum* primary forest; **, *P* < 0.01, *, *P* < 0.05, not significant (ns), *P* > 0.05.

**Figure 6:**
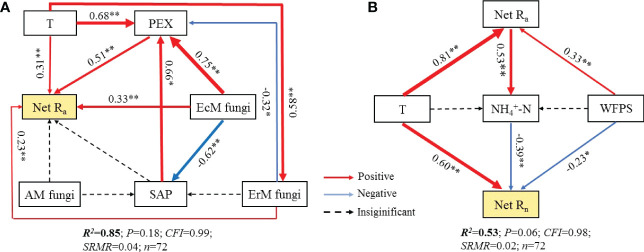
Structural equation models (SEM) analysis depicting the effects of soil PEX (peroxidase) activity, soil fungal functional guilds and soil environment key factors on soil net ammonification rate (Net R_a_) **(A)** and soil net nitrification rate (Net R_n_) **(B)**. T, temperature; WFPS, water-filled pore space. EcM fungi, ectomycorrhizal fungi; AM fungi, arbuscular mycorrhizal fungi; ErM fungi, ericoid mycorrhizal fungi; SAP, undefined saprotroph.

**Figure 7:**
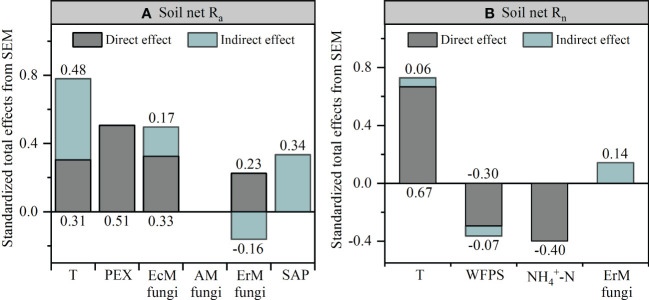
Standardized total effects of each variable on the soil net ammonification rate (Net R_a_) **(A)** and soil net nitrification rate (Net R_n_) **(B)** from the structural equation modeling (SEM) analysis. T, temperature; PEX, peroxidase; WFPS, water-filled pore space; EcM fungi, ectomycorrhizal fungi; AM fungi, arbuscular mycorrhizal fungi; ErM fungi, ericoid mycorrhizal fungi; SAP, undefined saprotroph.

Further research revealed that the difference in soil net R_a_ among three mycorrhizal forests was closely related to their soil environmental factors, soil PEX activity and soil fungal functional guilds (**Figures S2**, **6**, **7**). SEM analysis illustrated that soil temperature, the relative abundances of EcM fungi and ErM fungi had not only direct but also indirect effects on differential soil net R_a_ among three mycorrhizal forests. Similarly, SAP also indirectly affected the difference of soil net R_a_ among three mycorrhizal forests by regulating soil PEX activity (**Figures 6A**, **7A**).

Different from soil Net R_a_, soil environmental factors largely drove the difference found in soil Net R_n_ among three mycorrhizal forests. Soil temperature had a positive effect on the difference in soil net R_n_ among three mycorrhizal forests, while soil WFPS and soil $NH_{4}^{+}-N$ content had negative effect on it. We also found evidence of an indirect effect of soil net R_a_, by changing the soil $NH_{4}^{+}-N$ content, on the difference in soil net R_n_ among three forests (**Figures 6B**, **7B**).

## Discussion

### Seasonal patterns of soil net N mineralization

There were significant differences in seasonal variations of soil net N_min_ among three mycorrhizal forests in our research (**Figures 2A-C**). Temporal variation in forest soil net N_min_ was also suggested in previous studies (Zhao and Li, 2017; Xiao et al., 2022). Overall, our results indicated that soil mean net R_a_ and soil mean net R_n_ were positive in EcM forest and AM forest, while there were negative in ErM forest (**Figures 2D-F**). This implied the net production of $NH_{4}^{+}-N$ and $NO_{\;}3^{-}-N$ was dominant in EcM forest and AM forest, while the net consumption of $NH_{4}^{+}-N$ and $NO_{\;}3^{-}-N$ was dominant in ErM forest. This could be explained by the soil microbial in ErM forest absorbing more $NH_{4}^{+}-N$ and $NO_{\;}3^{-}-N$ to maintain the growth and its reproduction of the populations (Miller et al., 2009; Liu et al., 2021).

In our study, soil net R_a_ and soil net R_n_ in AM forest decreased gradually after July and June respectively (**Figures 2A, B**). This might be due to the decrease of soil temperature and increase in soil WFPS (**Figure 1**) which could inhibit soil microbial activity, slowing down soil net R_a_ and soil net R_n_ in AM forest (Guntiñas et al., 2012; Hishi et al., 2014). Simultaneously, our findings revealed that the effect of soil temperature on soil net ammonification rate was stronger than that of soil WFPS, while soil WFPS had a stronger effect on soil net nitrification rate than soil temperature in AM forest (**Table 2**). In contrast to AM forest, soil net R_a_ and soil net R_n_ in EcM forest and ErM forest showed trends of increasing in late autumn (**Figures 2A, B**). This was because soil net R_a_ and soil net R_n_ in EcM and ErM forests were not determined significantly by soil temperature. Increased soil WFPS promoted both soil net R_a_ in EcM forest and soil net R_a_ and soil net R_n_ in ErM forest (**Table 2**) which was consistent with previous study (Kou et al., 2018). This might be related to differing ecological tolerance strategies of soil microorganisms to soil temperature and soil WFPS among three mycorrhizal forests (Castro et al., 2010; Placell et al., 2012).

### Spatial (three mycorrhizal forests) effects on soil net N mineralization

Our results showed that ErM forest had the lowest soil net R_a_ and there was no difference between EcM forest and AM forest (**Figure 2D**). This might be due to a synergistic effect between soil temperature and different mycorrhizal plants (Tedersoo et al., 2014; Liu et al., 2017; Netherway et al., 2021). In our study, EcM forest had the highest relative abundance of EcM fungi, while the relative abundance of saprophytic fungi was lower (**Figure 4**). It had been confirmed that the relative abundance of soil EcM fungi increased with the abundance of EcM trees (Cheeke et al., 2017). Previous studies showed that host trees specificity could shape the relative abundances of soil fungi through co-evolution, niche differentiation, and niche conservatism (Tedersoo et al., 2013). EcM fungi have the mutualistic benefits with EcM trees (Velmala et al., 2013), which may be one of the reasons for the relatively high abundance of EcM fungi we found in EcM forest. Moreover, being the main functional groups of soil, EcM and saprophytic fungi would compete for SOC and N, and the strong competitiveness of EcM would limit the relative abundance of saprophytic fungi within the same ecological niche (Chen et al., 2022). Indeed, our results also showed that the relative abundances of soil AM fungi and saprotrophs in AM forests were higher than those in EcM forest and ErM forest (**Figure 4**), perhaps also due to the fact that the faster nutrient cycling pattern of AM forest (lower C: N, higher inorganic N content) was more suitable for the survival of AM fungi and saprophytic fungi (Bahram et al., 2020).

Our SEM results indicated that soil PEX activity has the strongest direct effect on soil net R_a_ (**Figures 6A**, **7A**). Previous studies showed that soil EcM fungi and SAP could secret PEX to degrade recalcitrant organic matter which could not be absorbed by plants and promote soil net R_a_ (Bödeker et al., 2014; Corrales et al., 2016; Ward et al., 2021). However, our results showed that ErM fungi were negatively correlated with soil PEX activity (**Figure 6A**) which might be due to microbial C limitation when soil C: N was low (Midgley and Phillips, 2014; Xu et al., 2022). As such, ErM fungi would reduce the energy available for synthase soil PEX (Carrara et al., 2018). At the same time, soil EcM fungi was superior to SAP in degrading soil organic N (Bahram et al., 2020). EcM fungi could compete with SAP to inhibit the relative abundance of SAP and thus affected soil net R_a_ (Bödeker et al., 2016; Argiroff et al., 2022). In addition, EcM fungi and ErM fungi could directly acquire low molecular weight organic matter (Read and Perez-Moreno, 2003). This should promote soil net R_a_ by reducing the products of decomposition processes in the first step of ammoniation (Levy-Booth et al., 2014). The SEM also revealed that soil temperature not only directly enhanced soil net R_a_, but also had a strong indirect effect (**Figures 6A**, **7A**). This principally arose *via* soil temperature which could affect soil PEX activity and the relative abundance of soil ErM fungi, thus affected soil net R_a_ (Tedersoo and Bahram, 2019; Fan et al., 2021).

Different from soil net R_a_, the SEM suggested that the difference of soil net R_n_ among three mycorrhizal forests was mainly determined by soil environmental factors (**Figures 6B**, **7B**). On the one hand, soil temperature was positively correlated with soil net R_n_, but negatively correlated with soil WFPS, a pattern consistent with other research findings (Borken and Matzner, 2009; Liu et al., 2017). On the other hand, soil temperature and WFPS promoted soil net R_a_ to produce more $NH_{4}^{+}-N$ which affected soil net R_n_. More recent studies also showed that soil $NH_{4}^{+}-N$ as a substrate would accelerate soil net R_n_ (Elrys et al., 2021). Yet we found that soil $NH_{4}^{+}-N$ content was negatively correlated with soil net R_n_ (**Figure 6B**). This discrepancy may be related to the net dynamics of soil $NO_{\;}3^{-}-N$ being dependent upon substrate content, as well as being affected by microbial absorption, N-fixation, and N-loss (Gao et al., 2015; Xiao et al., 2022).

In this study, SEM indicated that soil environmental factors and soil net R_a_ explained 53.7% of the difference of soil net R_n_ among three mycorrhizal forests (**Figure 6B**). However, soil fungal functional guilds had no effect on soil net R_n_ (**Figure 7B**). This might be due to soil net R_n_ was driven primarily by soil bacteria (Levy-Booth et al., 2014). Our results showed that AM forest soil also characterized by a high pH and a low C: N ratio (**Table 1**), which implied AM forest had a fast nutrient cycling (Phillips et al., 2013; Lin et al., 2017). It was reported that AM forest soil with less acidity (high pH) supported higher heterotrophic bacterial activity and greater potential for nitrification (Mushinski et al., 2020). Further, the nitrification pathway of bacteria was superior to that of fungi in soil with a low C: N ratio (Deng et al., 2018). In addition, AM forest had a higher ratio of bacteria to fungi than EcM forest and ErM forest, which also suggested that AM forest was more inclined to have soil net R_n_ driven by bacteria rather than fungi (Bahram et al., 2020).

## Conclusions

In this study, differences in soil net N_min_, soil fungal composition and soil enzyme activity among three mycorrhizal forests were highlighted. Notably, there were significant differences in soil net R_a_ and soil net R_n_ among three mycorrhizal forests. Further, soil temperature and soil WFPS differed in their effect on soil net R_a_ and soil net R_n_ in these three mycorrhizal forests. In response to the forest types, the difference in soil net R_a_ was determined mainly by soil temperature, soil fungal functional guilds and soil PEX activity. The difference in soil net R_n_ was related closely to soil temperature, soil WFPS and soil $NH_{4}^{+}-N$ content. Soil net R_a_ dominated the soil net N_min_ among three mycorrhizal forests. Overall, our results provide new insights into the mechanism of soil N dynamics in various mycorrhizal forests.

## Data availability statement

The raw data supporting the conclusions of this article will be made available by the authors, without undue reservation.

## Author contributions

MZ: investigation, data analysis, writing-original draft preparation, writing-review & editing. SL: methodology, investigation, writing-review & editing. XC: participated in the experiment, data analysis. MC: participated in the experiment, data analysis. JC: participated in the experiment, visualization. GX: Methodology. ZS: conceptualization, methodology, writing-review & editing. All authors contributed to the article and approved the submitted version.

## Supplementary material

Supplementary Figure 1 Rarefaction curve showing the sequence depth and observed OTUs. Note: EcM forest, *Abies fargesii* var. *faxoniana* primary forest; AM forest, *Cupressuschengiana* primary forest; ErM forest, *Rhododendron phaeochrysum* primary forest; -5, -7 and -11 represented May, July and November respectively.

Supplementary Figure 2 Correlations between the difference in soil net N mineralization rate, biological and abiotic factors. Note: PEX, peroxidase; POX, phenol oxidase; T, temperature; WFPS, water-filled pore space; AM fungi, arbuscular mycorrhizal fungi; EcM fungi, ectomycorrhizal fungi; ErM fungi, ericoid mycorrhizal fungi; SAP, undefined saprotroph; ‘Others’ include endophyte and lichenized. Pathotrophs include animal pathogen, plant pathogen and mycoparasites; Net R_a_, net ammonification rate; Net R_n_, net nitrification rate; **, *P*< 0.01; *, *P*< 0.05; not significant (ns), *P* > 0.05.
