# Supplementary figures and images for: The effects of ectomycorrhizal and saprotropic fungi on soil nitrogen mineralization differ from those of arbuscular and ericoid mycorrhizal fungi on the eastern Qinghai-Tibetan Plateau
Source: Front Plant Sci. 2023 Jan 4;13:1069730. doi: 10.3389/fpls.2022.1069730 (PMC9846110; doi:10.3389/fpls.2022.1069730)

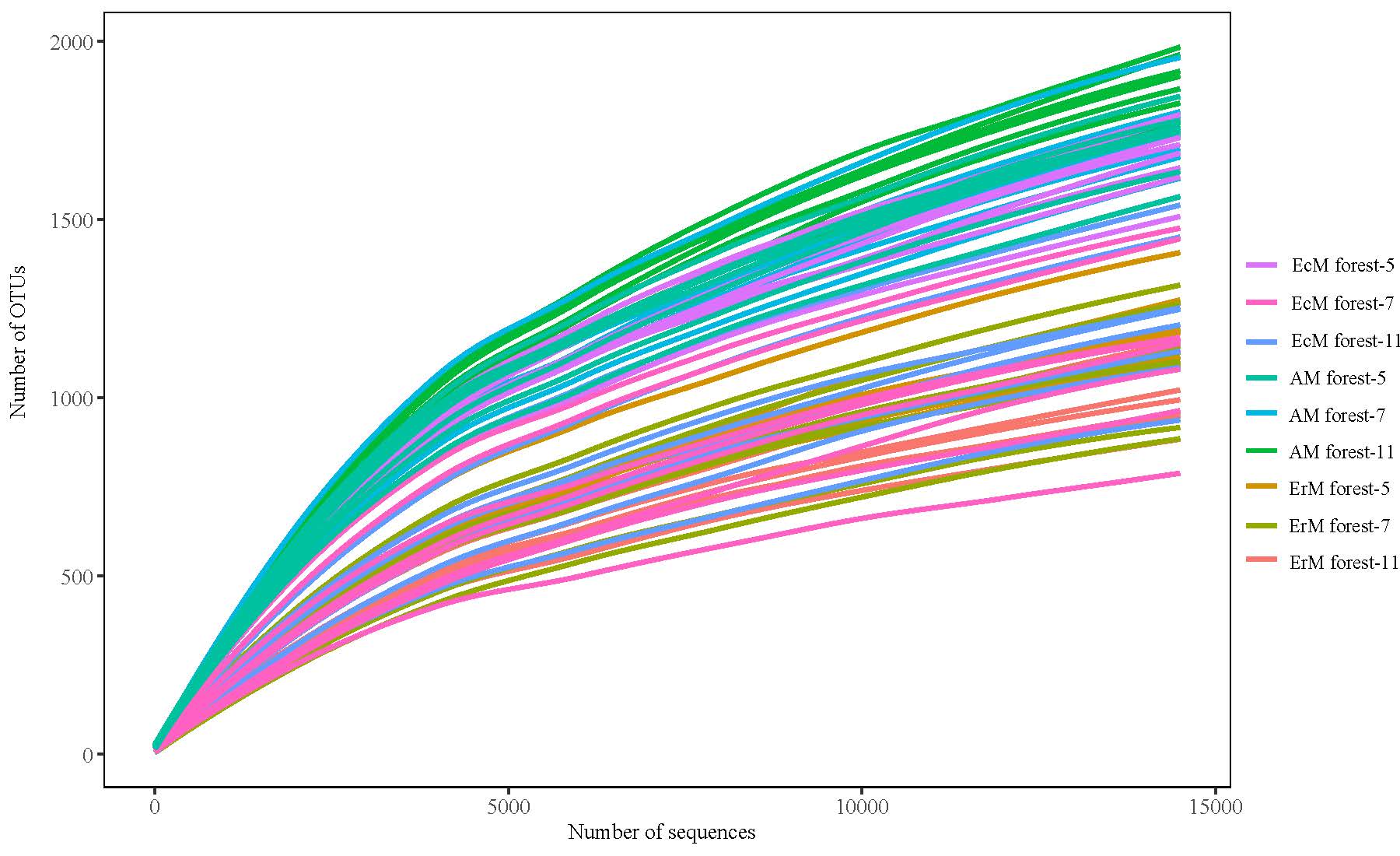

Supplement: Supplementary Figure 1 — Rarefaction curve showing the sequence depth and observed OTUs. Note: EcM forest, Abies fargesii var. faxoniana primary forest; AM forest, Cupressuschengiana primary forest; ErM forest, Rhododendron phaeochrysum primary forest; -5, -7 and -11 represented May, July and November respectively. [file Image_1.jpeg]

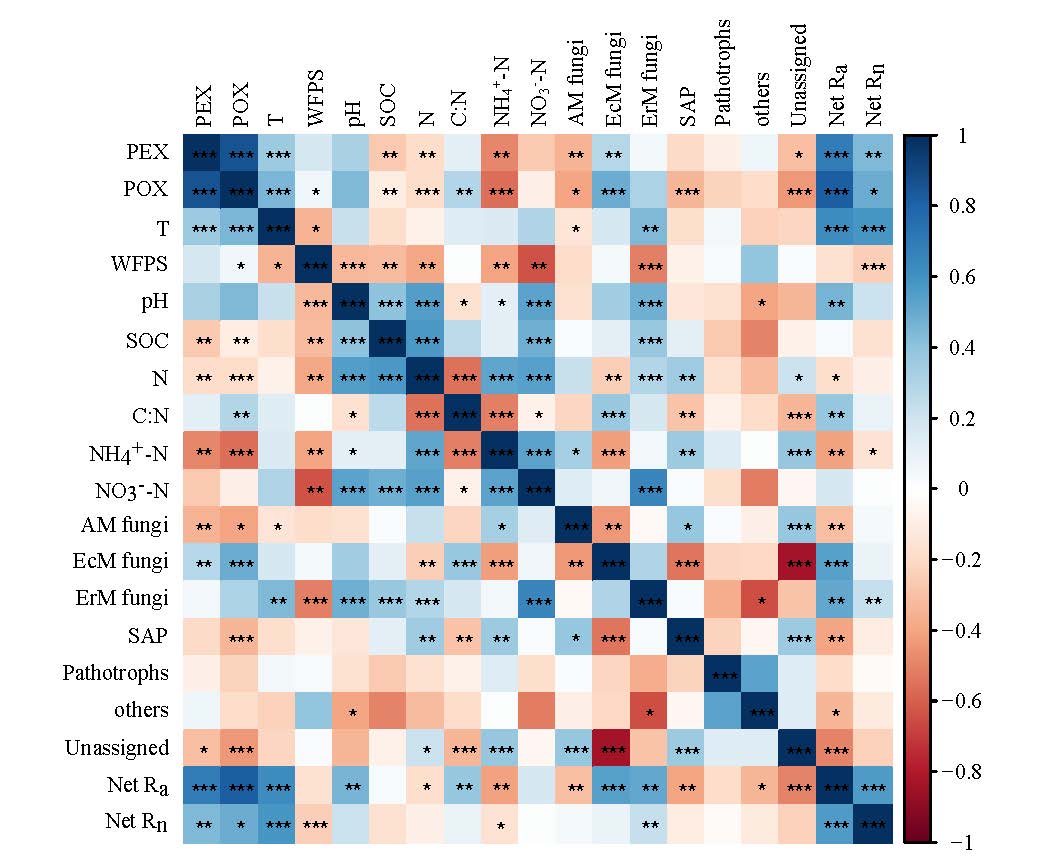

Supplement: Supplementary Figure 2 — Correlations between the difference in soil net N mineralization rate, biological and abiotic factors. Note: PEX, peroxidase; POX, phenol oxidase; T, temperature; WFPS, water-filled pore space; AM fungi, arbuscular mycorrhizal fungi; EcM fungi, ectomycorrhizal fungi; ErM fungi, ericoid mycorrhizal fungi; SAP, undefined saprotroph; ‘Others’ include endophyte and lichenized. Pathotrophs include animal pathogen, plant pathogen and mycoparasites; Net Ra, net ammonification rate; Net Rn, net nitrification rate; **, P< 0.01; *, P< 0.05; not significant (ns), P > 0.05. [file Image_2.jpeg]
